# Susceptibility of virulent and resistant *Escherichia coli* strains to non-polar and polar compounds identified in *Microplumeria anomala*

**DOI:** 10.14202/vetworld.2020.1376-1387

**Published:** 2020-07-21

**Authors:** Livia Roberta Piedade Camargo, Vania Maria de Carvalho, Ingrit Elida Collantes Díaz, Mateus Luís Barradas Paciencia, Sergio Alexandre Frana, Riad Naim Younes, Antonio Drauzio Varella, Luiz Fernando Lima Reis, Ivana Barbosa Suffredini

**Affiliations:** 1 Graduate Program in Environmental and Experimental Pathology, Paulista University, São Paulo, Brazil; 2 Department of Chemistry Engineer, Chemistry and Textile Engineer Faculty, Engineer National University, Lima, Peru; 3 Center for Research in Biodiversity, Paulista University, São Paulo, Brazil; 4 São José Hospital, São Paulo, Brazil; 5 Education and Research Center, Sírio-Libanês Hospital, São Paulo, Brazil

**Keywords:** antibacterial agents, companion animals, livestock, plant extracts, poultry, tropical rainforest

## Abstract

**Background and Aim:**

*Escherichia coli* is one of the main pathogens responsible for veterinary and human infections, and it is associated with significant economic losses in the livestock, as it causes severe diseases to humans, particularly in children. For that reason, there is a need for introducing new drugs to treat *E. coli* diseases. The Brazilian species richness is a source of potential new antibacterial natural products. The study aimed at the biological and chemical investigation of the organic extract obtained from the stem of *Microplumeria anomal*a (Apocynaceae), EB127, as it was identified as a potential source of new antibacterial compounds to be used in Veterinary.

**Materials and Methods:**

The antibacterial activity was evaluated by disk diffusion and microdilution assays; chromatography, nuclear magnetic resonance spectrometry, and mass spectrometry were used in the isolation and identification of compounds.

**Results:**

EB127 showed activity against *E. coli* ATCC25922, and against three *E. coli* strains that were isolated from frigarte’s cloaca, named 31/1A, 35A, and 51A. Lupeol, 3-acetyl-11-oxo-β-amyrin, 3-acetyl-11-oxo-α-amyrin, sitosterol, stigmasterol, 3β,7α-dihydroxy-cholest-5-ene, 3β-hydroxy-cholest-5-en-7-one, and 3β-hydroxy-cholest-5,22-dien-7-one were identified in fraction Hex/CHCl_3_, while loganin, loganic acid, methylanomaline, and anomaline were all identified in EB127 and protocatechuic acid hexoside, ferulic acid, secoxyloganin, feruloylquinic acid, vanillic acid hexoside, protocatechuic acid-4-O-β-hexoside, and rosmarinic acid were tentatively identified in fraction 10%ACN/H_2_O. *E. coli* 51A (virulent/non-resistant) showed sensitivity to the antibacterial action of fraction Hex/CHCl_3_ which contains alkaloids, triterpenes, and steroids, while *E. coli* 35A (resistant/non-virulent) were more susceptible to 10%ACN/H_2_O, which contains iridoids as loganin and loganic acid, and glycosylated and non-glycosylated caffeic acids.

**Conclusion:**

Fraction 10%ACN/H_2_O is of interest in pursuing new drugs to treat resistant *E. coli*, in veterinary. All compounds were isolated from the plant for the first time and have shown potential as new antibacterial natural products from Amazon plants to be used in veterinary and human diseases.

## Introduction

*Escherichia coli* is one of the most important micro-organisms in veterinary pathology due to its impact on animal production and in pet’s disease. *E. coli* is a Gram-negative bacterium with a short rod shape, facultative anaerobe that does not sporulate [1]. Its usual habitat is the gastrointestinal tract in most of the homeothermic animals and for that reason can be found in fecal waste [1]. As a pathologic agent, the bacterium is responsible for diseases that affect production animals, resulting in bloody-liquid diarrhea [2], eventually leading to neurological alterations, anemia, and/or colitis in small ruminants, swine and in livestock [1]; it may also cause vascular lesions in swine, and hemorrhage in calves. The bacterium can affect breeding farm animals causing impairment of the respiratory tract, leading to the reduction of egg posture, salpingitis and to an eventual embryonic loss, colibacillosis in calves, piglets, lambs, foals and in birds [3] is also reported, as well as mastitis in cows [1]. *E. coli* also plays an important role in diseases affecting pets, particularly causing pyometra and cystitis in female dogs and cats which are the most frequent disease reported in veterinarian offices [4]. Not only is the economic impact on agricultural production extremely relevant but also the emotional/financial impact on pet owners.

*E. coli* is one of the most important known pathogen organisms, both for livestock and men. In the present work, one standard strain of *E. coli* and three isolates from frigate cloaca having different levels of virulence and resistance were used in the antibacterial activity of *M. anomala* extract. One of the main concerns related to the treatment of bacterial pathogenic diseases is the resistance acquired by the micro-organism to fight antibiotics. Resistance can be defined as the ability of micro-organism to grow in an environment containing high amounts of antibiotics, no matter how long the treatment spans [5,6], and the access of resistant strains into the study is a *sine qua non* condition to investigate the antibacterial profile of a new treatment proposal. For that reason, the three virulent and/or resistant *E. coli* isolates that were obtained from sea birds in previous study [7] and were gently provided by the authors to be used in the present work. Birds have the ability to rapidly disseminate *E. coli*, a situation that can lead to the contamination of water reservoirs and food sources [8]. As a consequence, birds also can be considered as biological indicators of environmental pollution [9]. As the dissemination of the pathogen can culminate in the elevation of the number of cases of diarrheal diseases among children, particularly in developing countries as Brazil [10] and others [11], and among production and company animals, it is imperative that new drugs to treat diseases decurrent of such pathogen are introduced in therapy protocols. Antibiotics are currently being successfully used as an important tool against veterinary and human infections. Antimicrobials from natural products are important alternative to be effectively considered in the infection treatments and may play a significant role in therapeutic strategies. The prospection of natural products from plants has been one of the main strategies adopted for the identification of new antimicrobial drugs. Brazil is the country with high biodiversity, containing 20% of all species in the world; the Amazon Rain Forest contains about 17% of all Brazilian species, which makes the forest one of the Brazilian hotspots in terms of species richness [12]. Thus, research related to the identification of new pharmacologically active molecules from Brazilian plants to be introduced as new tools to combat *E. coli* diseases is of paramount importance.

The study aimed at the biological and chemical investigation of the organic extract obtained from the stem of *Microplumeria anomala* (Apocynaceae), EB127, as it was identified as a potential source of new antibacterial compounds to be used in Veterinary. The extract was previously selected in a high-throughput screening [13] to be chemically and biologically studied, and to have active compounds identified. *M. anomala* is the only species within its genus, *Microplumeria* Baill. (heterotypical synonym is *Cylindrosperma* Ducke.), and it is also known as *Microplumeria sprucei* Baill. Despite being native to our forests, it is not endemic in Brazil. It is commonly found in the igapó *forests*, which is found in the states of Amazonas and Pará; its basyonim is *Aspidosperma anomalum* Müll. Arg., its homotypical synonym is *Cylindrosperma anomalum* (Müll. Arg.) Ducke, and its heterotypical synonym is *M. sprucei* Baill. The species can also be found in Colombia and Venezuela Amazon forests.

## Materials and Methods

### Ethical approval

The present study was performed with standard *Escherichia coli* strain ATCC29212 and with isolates from *Fregata magnificens*, which were obtained for a previous study by Saviolli and colleagues [7], who provided the strains to be used in the current study. The authors of the past study have kept the strains in the freezer as a backup for further studies, among which, the present one. In the former study, the authors described how the strains were obtained and kept, and also includes the authorization that was then obtained for their study, which is in accordance to the Research Ethics Committee (Permit Number: 1368/2008; Veterinary Faculty/ São Paulo University, in compliance with federal permits issued by the Brazilian Ministry of Environment Permit Numbers 2997/1 and 16553–1).

### Plant collection and extract preparation

The stem of *M. anomala* was collected in Manaus, AM, Brazil, latitude 3°5’36” longitude 60°26’28”, in *igapó* forest, under licenses # 14895-6 (ICMBio/MMA/Brazil) and 12A/2008 (IBAMA/CGen/MMA). The collection was carried out in the Amazon rain-forest, the species was determined by Dr. Alexandre Adalardo de Oliveira, and the voucher deposited at Herbarium UNIP. A voucher was deposited at UNIP Herbarium [P.S.C., 136 (UNIP)]. After being dried under 40°C and ground in a hammer mill (Thomas, USA), the plant material was submitted to a 24h-maceration with methanol and dichloromethane (1:1) (solvents, Merck, Germany). The organic extract, named EB127, (2.55% yield) had the solvents evaporated under reduced pressure (Buchi) before being stored at −27°C (Revco) until its use [14]. EB127 was diluted with 50% dimethyl sulfoxide (DMSO 50, Synth, Brazil) in water to the concentration of 200 mg/mL for their use in the biological assays. Chlorhexidine 1% was used as a reference drug [15,16]. The material used in this study was endotoxin-free.

### Bacterial strains

*E. coli* (Migula) Castellani and Chalmers ATCC^®^ 25922™ (ATCC, USA) and strains 31/1A, 35A, and 51A, obtained from frigate (*Fregata magnificens*) cloaca, were used in the biological assays. Isolated bacteria were previously classified according to their virulence/resistance phenotypes (Table-1) [7].

**Table-1:** Pathogenicity of three strains of *Escherichia coli* collected from the frigate (*Fregata magnificens*) cloaca.

Virulence genes	*Escherichia coli* strains		

	31/1A or (O2:H7)	35A or (NP)	51A or (ONT:H7)
papC	+	-	+
fyuA	+	-	+
iucD	+	-	+
ibeA	+	-	-
malX	+	-	+
traT	+	-	+
colV	+	-	+
iroN	+	-	+
ompT	+	-	+
hlyF	+	-	+
iss	+	-	+
iutA	+	-	+
Resistance to antibiotics	Amp., Amo., Cef.	Amp., Amo., Tet., Fluor., Sulfa.+Trim.	X

Amo.=Amoxicillin, Cef=Cephalexin, Amp.=Ampicillin, Tet.=Tetracycline, Fluor.=Fluoroquinolone, Sulfa.=Sulfamethoxazole, Trim.=Trimethoprim;+=Presence of virulence gene, −=Absence of virulence gene, X=Sensitivity to many antibiotics. O2:H7, NP, and ONT:H7 were the names of the *E. coli* isolates as previously described [7]

### Disk diffusion assay (DDA)

All *E. coli* strains were individually cultivated and tested under the same growth conditions, using Müeller-Hinton agar (MHA; Oxoid^®^, Thermo, USA) Petri dishes (JB Labor, Brazil). Fresh bacteria suspensions were prepared at 0.5 McFarland (or 1.5 × 10^8^ CFU/mL). Petri dishes containing 15 mL of MHA, prepared according to the manufacturer’s instructions, were surface-inoculated [17] using sterile swabs, to perform DDA. Hence, six sterile 6 mm diameter paper disks were set at equidistant points on the agar surface. After that, 10 μL of the sample to be tested was placed on a disk. Petri dishes were put in an incubator at 36°C for 24 h. Finally, the inhibition zone diameters were measured with an electronic caliper rule.

The assays were performed with EB127, its residues and fractions, as well as with the isolated ­substances, in triplicate, in Petri dishes of 120 mm diameter. Two diameters of each inhibition zone generated were measured, so, six measurements were obtained for each treatment. The same procedure was repeated for all analyzes.

### Microdilution broth assay (MBA)

The extract and its fractions were tested in the MBA; Oxoid, London, England), using sterilized Müeller-Hinton broth medium (MHB; Oxoid^®^, Thermo, USA), in 96-well microplates (JB Labor, Brazil), with inoculum adjusted to a concentration of 1 × 10^8^ colony-forming unit per mL (CFU/mL) grown on sterilized Müeller-Hinton agar medium (MHA; Oxoid^®^, Thermo, USA). A 190 μL aliquot of the inoculum was dispensed into each well, and 10 μL aliquots of extract/fractions were added to the inoculum. Microplates were incubated at 36°C for 24 h. Inhibition of bacterial growth was assessed by subculturing each well of the microplate on sterile agar medium, so as to evaluate bacterial growth. Following a procedure similar to that described above in sterile MBA, minimal inhibitory concentration (MIC) and minimal bactericidal concentration (MBC) were obtained for the samples, against the four *E. coli* strains. Solvent dimethyl sulfoxide (DMSO, Merck, Germany) were tested as vehicle control [16,18].

### Cytotoxicity assay

The cytotoxicity was tested by the sulforhodamine B (dye, Sigma, USA) assay against human breast carcinoma (MCF-7) and prostate carcinoma (PC-3) cell lines [19-21]. The test was performed for the crude extract and its purified fractions. The cells were grown in RPMI 1640 (culture medium, Bio Whittaker, USA) medium with 10% fetal bovine serum and 1% *L*-glutamine (Sigma, USA), for 24 h. The samples were dissolved in DMSO-H_2_0 (1:1 v/v) and tested, in sextuplicates, against positive control doxorubicin (Sigma, USA).

### Fractionation of active extract

**Figure-1:**
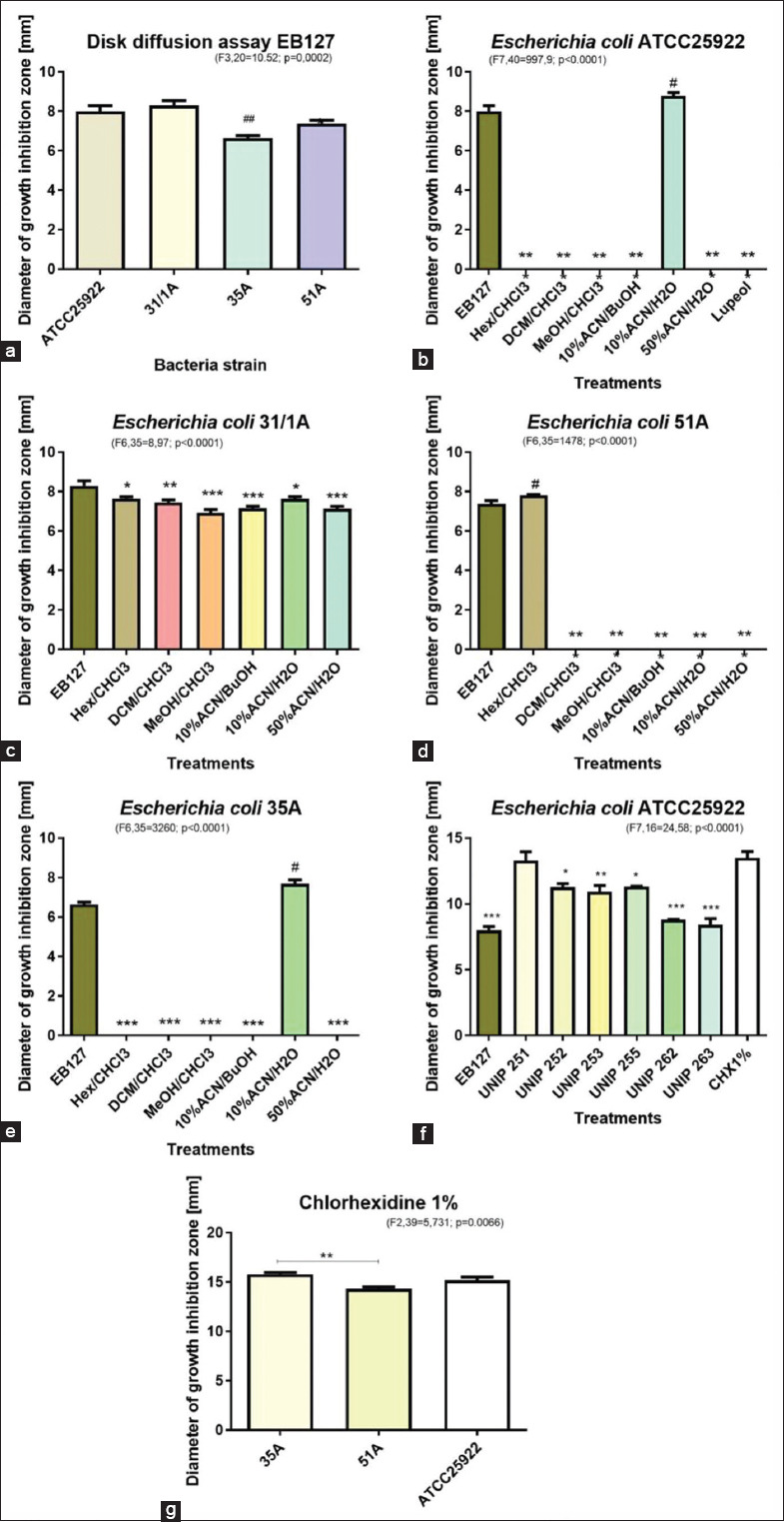
(a-g) Fractionation of the crude extract EB127 obtained from the stem of *Microplumeria anomala* using liquid-liquid partition and chromatography techniques in C-18 and Sephadex LH-20 columns, which resulted in nine fractions. CHCl_3_=Chloroform, BuOH=Butanol, CC=Column chromatography, Hex=Hexane, DCM=Dichloromethane, MeOH=Methanol, ACN=Acetonitrile; H_2_O=Water.

EB127 was fractionated using a liquid-liquid partition system (Figure-1), as described first [18]. The extract was resuspended in 90% methanol (MeOH) and was added to a glass column, to be subjected to subsequent extractions with solvents CHCl_3_, BuOH, and water, which resulted in three residues named CHCl_3_, BuOH, and H_2_O, respectively. Organic residues had the solvents evaporated (Buchi) while the aqueous fraction was lyophilized (Virtis). The residue CHCl_3_ was subjected to a new fractionation using column chromatography (CC) packed with Sephadex LH-20. The solvents hexane (Hex), dichloromethane (DCM), and MeOH were subsequently used as the mobile phase, to obtain three new fractions, as shown in Figure-1. Both residues named residue BuOH and residue H_2_O were subjected to CC packed with C-18 reversed-phase silica as the stationary phase and the mixture composed of acetonitrile (ACN):water (H_2_O) acidified with 0.1% trifluoroacetic acid (TFA, Synth, Brazil) in the proportion of 10% and 50%, and MeOH100% as the subsequent mobile phases to perform the elution. Three new fractions were obtained for each residue and were named Hex/CHCl_3_, DCM/CHCl_3_, MeOH/CHCl_3_, 10%ACN/BuOH, 50% CAN/BuOH, MeOH/BuOH, 10%ACN/H_2_O, 50% CAN/H_2_O, and MeOH/H_2_O (solvents, Merck, Germany).

### High-performance liquid chromatography (HPLC)

Reversed-phase HPLC fingerprints were obtained for fractions MeOH/CHCl_3_, 10%ACN/BuOH, 50%ACN/BuOH, MeOH/BuOH, 10% ACNH_2_O, 50%ACNH_2_O, and MeOH/H_2_O (solvents, Merck, Germany). Each sample was weighed (1 mg), solubilized in MeOH (2 mL) and filtered with 0.45 μM Millex™ JBR13LCR1 filters (Millipore, USA). The eluent system was A: 0.1% TFA water, B: HPLC grade ACN, filtered with 47 mm diameter Nylon (0.45 μM) Phenex Filter Membranes (AFO-0504; Phenomenex, USA) filter. The chromatographic run was performed in gradient system: T0 → 5 = 5% B, T5 → 35 = 5% B up to 100% B, T35 → 40 = 100% B, T40 → 50 = 100% B up to 5 % of B, T50 → 55 = 5% of B, in Agilent’s HPLC (1220LL Systems, with 254 nm UV detector, Agilent, USA) and DAD detector (Agilent 1260DAD, Agilent, USA), injection volume was 20 μL.

### Liquid-chromatography coupled to mass spectrometer (LC-MS)

Fraction 10%ACN/H_2_O was submitted to analysis performed in high-performance liquid chromatography coupled to electrospray ionization mass spectrometer (HPLC-MS; Bruker Daltonics Esquire 3000, Bruker, United Kingdom). Equipment was set at T0-10 = 5% ACN, T10-11 = 10% ACN, T11-20 = 10% ACN, T20-21 = 20% ACN, T21-40 = 20% of ACN, T40-41 = 27% of ACN, T41-60 = 27% of ACN, T60-61 = 32% of ACN, T61-68 = 32% of ACN, T68-69 = T69-85 = 39% ACN, T85-86 = 50% ACN, T86-96 = 50% ACN, T96-106 = 100% ACN, T106-110 = 100% ACN, and T110-120 = 5 % ACN.

### Hydrogen and carbon nuclear magnetic resonance (NMR)

The purified fractions were subjected to hydrogen and carbon NMR spectrometry in Bruker AIII spectrometer (^1^H-NMR, 300 MHz, CDCl_3_; ^13^C NMR, 75 MHz, CDCl_3_, respectively) analyses to carry out structural elucidation of the samples.

### Statistical analysis

Statistical analyses were performed by one-way analysis of variance, with Dunnett’s multi-comparison post-test. Statistical differences were considered at a significant level of α < 0.05 (GraphPad Prism 5.0, USA).

## Results

Figure-1a shows the results related to the antimicrobial activity in the DDA of EB127 against the four strains of *E. coli*. The strains that were used in the assays are displayed in Table-1. Strain 35A, which shows resistance to antimicrobials and is not virulent, showed the lower diameter of growth inhibition zone when compared to the effect of EB127 observed to the other three strains (F_(3,20)_=10.52; p=0.0002). Figure-1b reports the diameter of the growth inhibition zone formed by EB127 and its fractions against *E. coli* ATCC25922. There was a clear effect of fraction 10%ACN/H_2_O, which was as effective as EB127, in relation to the other treatments (F_(7,40)_=997.9; p<0.0001). Figure-1c shows the diameter of growth inhibition zone formed by EB127 and its fractions against *E. coli* 31/1A, a virulent and relatively resistant *E. coli* strain. Although there was significance in the growth inhibition zone of EB127 in relation to the fractions (F_(6,35)_=8,97; p<0.0001), it is possible to observe that fractions Hex/CHCl_3_ and 10%ACN/H_2_O were more active than the other ones. Figure-1d shows the diameter of growth inhibition zone formed by EB127 and its fractions against *E. coli* 35A, a non-virulent but resistant *E. coli* strain. Fraction 10%ACN/H_2_O was significantly more active than EB127 or the other fractions (F_(6,35)_=3260; p<0.0001). Figure-1e shows the diameter of growth inhibition zone formed by EB127 and its fractions against *E. coli* 51A, a virulent but not resistant *E. coli* strain. Fraction Hex/CHCL_3_ was significantly more active than EB127 or the other fractions (F_(6,35)_=1478; p<0.0001). Figure-1f reports the diameter of growth inhibition zone formed by EB127 and some purified fractions against *E. coli* ATCC25922. Although all purified fractions performed better than EB127, fraction UN-251 showed to be as active as CHX 1% (F_(7,16)_=24.58; p<0.0001).

EB127 was tested in the MBA model to determine MIC and MBC against the four different *E. coli* strains which suspensions were prepared at different concentrations. Reports are displayed in Table-2. Furthermore, EB127 and its total alkaloid fraction (TA) were submitted to a cytotoxic assay, which results are shown in Table-3. None of the tested samples were cytotoxic in this model, when compared to doxorubicin.

**Table-2:** MIC and MBC obtained from the action of EB127 against four strains of *Escherichia coli* at concentrations of 1.5 × 10^2^, 1.5 × 10^3^ and 1.5 × 10^4^ UFC/mL.

Bacterial strain	1.5 × 10^2^ UFC/mL	1.5 × 10^3^ UFC/mL	1.5 × 10^4^ UFC/mL
ATCC25922	MIC=MBC=300 mg/mL	MIC=MBC=300 mg/mL	MIC=MBC=300 mg/mL
31/1A	MIC=MBC=200 mg/mL	MIC=MBC=200 mg/mL	MIC=MBC=500 mg/mL
35A	MIC=MBC=300 mg/mL	MIC=MBC=300 mg/mL	MIC=MBC>1000 mg/mL
51A	MIC=MBC=500 mg/mL	MIC=MBC>1000 mg/mL	MIC=MBC>1000 mg/mL

MIC=Minimum inhibitory concentration, MBC=Minimum bactericidal concentration

**Table-3:** Results obtained from the cytotoxicity analyses of EB127 and its total alkaloid fraction (TA) against breast (MCF-7) and prostate (PC-3) human cancer cell lines.

	% Growth	% Growth inhibition	% Lethality
Breast (MCF-7)			
DOXO	-	-	25.71
DMSO	217.42	0.00	0.00
EB127	78.01	21.99	0.00
TA	101.60	0.00	0.00
100%TA	98.11	1.89	0.00
50% TA	100.86	0.00	0.00
25% TA	85.27	14.73	0.00
15% TA	90.54	9.46	0.00
11% TA	96.22	3.78	0.00
7% TA	97.08	2.92	0.00
Prostate (PC-3)			
DOXO	-	-	16.27
DMSO	199.38	0.00	0.00
EB127	78.09	21.91	0.00
TA	88.09	11.91	0.00
100%TA	94.70	5.30	0.00
50% TA	100.81	0.00	0.00
25% TA	96.07	3.93	0.00
15% TA	95.70	4.30	0.00
11% TA	105.55	0.00	0.00
7% TA	100.31	0.00	0.00

DOXO=Doxorubicin, DMSO=Dimethyl sulfoxide, TA=Total alkaloid fraction

**Figure-2:**
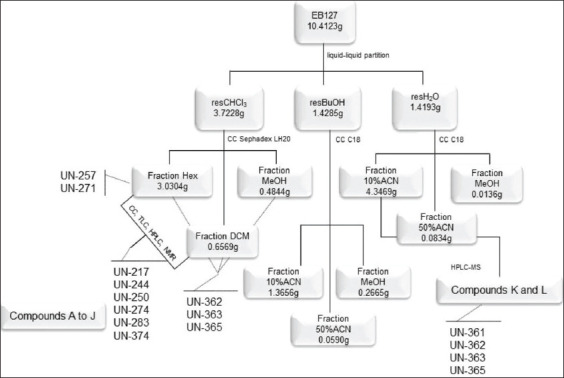
Compounds isolated and identified from EB127, obtained from *Microplumeria anomala*. A=Lupeol, B=3-acetyl-11-oxo-β-amyrin, C=3-acetyl-11-oxo-α-amyrin, D=Sitosterol, E=Stigmasterol, F=3β,7α-dihydroxy-cholest-5-ene, G=3β-hydroxy-cholest-5-en-7-one, H=3β-hydroxy-cholest-5,22-dien-7-one, I=Methylanomaline, J=Anomaline, K=Loganin, L=Loganic acid.

**Figure-3:**
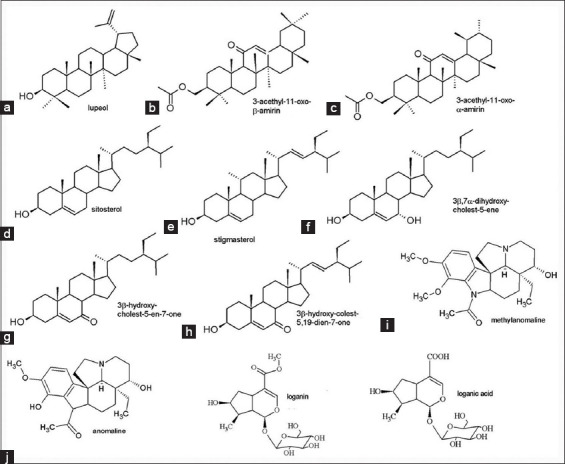
Results obtained from the agar diffusion assay, in which EB127 obtained from *Microplumeria anomala* was tested against four strains of *Escherichia coli* named ATCC25922, 31/1A, 35A, and 51A. All strains but ATCC were isolated from seabird cloaca. One-way ANOVA followed by Dunnett’s post-test was adopted (significance at α < 0.05). (a) Disk diffusion assay (DDA) of EB127 against four *E. coli* strains; (b) DDA of EB127 and its fractions against *E. coli* strain ATCC25922. (c) DDA of EB127 and its fractions against *E. coli* strain 31/1A. (d) DDA of EB127 and its fractions against *E. coli* strain 35A. (e) DDA of EB127 and its fractions against *E. coli* strain 51A. (f) DDA of EB127 and its purified fractions against *E. coli* strain ATCC25922. (g) Chlorhexidine 1% evaluation of antimicrobial activity against *E. coli* strains.

Figure-2 shows the fractionation EB127 and the molecules that were isolated and identified in *Microplumeria anomala*. Figure-3 shows the molecular structures of the compounds that were isolated from the extract EB127, obtained from *Microplumeria anomala*.

### Isolated compounds

Compounds had the structure elucidated based on data obtained from hydrogen and carbon NMR and from mass spectrometry.

#### Lupeol

Lupeol (A) was identified in two fractions called UN-217 (10 mg) and UN-250 (9.8 mg). The 1H-NMR spectrum (CDCl_3_) shows the characteristic signals of a triterpene, such as two doublets in δ 4.69 and 4.57 with *J =* 4.69Hz, both doublets corresponding to the hydrogens bound to C-29, a double-doublet in δ3, 19 with *J =* 10.94, 5.14 Hz corresponding to H-3. Six singlets between δ0.76 and 1.035 corresponding to the methyl groups 23, 24, 25, 26, 27, and 28 and a singlet at δ 1.69 of methyl C-30. The ^13^C-NMR spectrum (CDCl_3_) shows 29 carbons: 14,52 (C-27), 15,35 (C-24), 15,95 (C-26), 17,98 (C-28), 18.29 (C-6), 19,28 (C-30), 20,90 (C-11), 25,10 (C-12), 27,38 (C-15), 27,42 (C-2), 27,96 (C-23), 29.82 (C-21), 34,25 (C-7), 35,55 (C-16), 37,14 (C-10), 38,02 (C-13), 38,68 (C-1), 38,83 (C-4), 39.97 (C-22), 40,79 (C-8), 42,80 (C-14), 42,97 (C-17), 47,95 (C-19), 48,27 (C-18), 50,41 (C-9), 55.27 (C-5), 78,97 (C-3), 109,30 (C-29), and e 150,93 (C-20), data were compared with the literature [22].

#### 3-Acetyl-11-oxo-β-amyrin and 3-acetyl-11-oxo-α-amyrin

The mixture of 3-acetyl-11-oxo-β-amyrin (B) and 3-acetyl-11-oxo-α-amyrin (C) was identified in the sample UN-257 = 10.1 mg. The ^1^H-NMR spectrum (CDCl_3_) shows the characteristic signals of an acetylated oleane and ursane skeleton such as the singlets at δ 5.69 and 5.55, each corresponding to the H-12 of the ursane and oleane skeleton, respectively. A double-doublet sign at δ 4.52 with *J =* 11.79 and 4.61 Hz can be seen and corresponds to the H-3 of both skeletons. The singlet signs at δ 2.36 and 2.35 corresponds to the H-9 of the ursane and oleane skeleton, respectively. A singlet sign at δ 2.06 corresponds to methyl acetate. Also, the 14 singlets ranging from δ 0.82 to 1.37 correspond to the methyl groups of both compounds, and the two doublets at δ 0.81 and 0.90 with *J =* 6 Hz corresponds to the methyl groups attached to the C-19 and C-20 of the ursane skeleton. The ^13^C-NMR spectrum (CDCl_3_) reported for 11-oxo-3-β-methylamine shows the following signs: 16.39 (C-25), 16.67 (C-24), 18.49 (C-6), 21.29 (CH 3 -COO), 24.47 (C-30), 23.55 (C (C-15), 27.21 (C-2), 28.04 (C-23), 28.74 (C-28), 30 (C-21), 34.42 (C-21), 36.48 (C-20), 36.48 (C- 23), 36.91 (C-10), 38.03 (C-4), 38.78 (C-1), 43.38 (C-8), 45.10 (C- 13 (C-14), 47.59 (C-18), 54.97 (C-5), 61.66 (C-9), 80.64 (C-3), 128.04 (C-13), 171.00 (COO), and 200.17 (C-11). ^13^C-NMR spectrum (CDCl_3_) and for 11-oxo-3α-methylamine, the following signs: 16.54 (C-24 and C-25), 17.45 (C-26), 18.69 (C-6), 20.47 (C-30), 21.29 (CH 3 COO), 23.36 (C- 27), 27.48 (C-2), 28.04 (C-23), 28.48 (C-15) (C-10), 38.03 (C-7), 36.78 (C-10), 38.03 (C-7), 38.86 (C-1), 39.19 (C-20), 39.26 (C-19), 40.89 (C-22), 43.62 (C-8), 45.13 (C (C-3), 130.37 (C-12), 164 (C-3), 99 (C-13), 171.00 (COO), and 199.71 (C-11). The data were compared with the literature [23].

#### Sitosterol and stigmasterol

The mixture of sitosterol (D) and stigmasterol (E) was identified in the sample UN-271 = 17.5 mg. The 1H-NMR spectrum (CDCl_3_) showed the characteristic signals of these steroids as two broad singlets at δ 5.36 and 5.35 corresponding to the stigmasterol and sitosterol H-6, respectively, a double-doublet at δ 5.16 with *J =* 15.22, 8.8 Hz corresponding to the H-20 of stigmasterol, a double-doublet at δ 5.02 with *J =* 15.05, 8.62 Hz corresponding to the H-19 of stigmasterol, and a multiplet in δ 3.53 corresponding to the H-3 of both steroids. The signals corresponding to the steroid methyl groups are present in δ 0.69-1.26. The ^13^C-NMR spectrum (CDCl_3_) for sitosterol: 11.85 (C-29), 11.96 (C-18), 18, (C-19), 19.79 (C-27), 21.07 (C-11), 23.04 (C-28)), 24.28 (C-15), 26.06 (C-25), 28.23 (C-16), 29.68 (C-23), 31.63 (C-2), 31.88 (C-7 and C-8), 33.93 (C-22), 36.13 (C-20), 36.16 (C-10), 37.23 (C-1), 39.75 C-12), 42.19 (C-13), 42.28 (C-4), 50.13 (C-9 and C-24), 56.04 (C-17), 56.74 (C-6), and C (C-5), for stigmasterol, 12.02 (C-29), 12.23 (C-18), 18.97 (C-26), 19.38 (C-19), 21.07 (C-11), 21.20 (C-21 and C-27), C-15), 25.39 (C-28), 28.89 (C-16), 31.44 (C-2), 31.88 (C-7, C-8 and C-25), 36 (C-10), 37.23 (C-1), 39.66 (C-12), 40.47 (C-20), 42.19 (C-13), 42.28 (C- 4), 50.13 (C-9), 51.22 (C-24), 55.93 (C-17), 56.85 (C-14), 71.78 (C- (C-6), 129.25 (C-23), 138.31 (C-22), and 140.73 (C-5), data were compared with the literature [24].

#### 3β,7α Dihidroxy-colest-5-en

Sample UN-274 = 5.3 mg contained the steroid 3β,7α-dihydroxy-cholest-5-ene (F). The ^1^H-NMR spectrum (CDCl_3_) shows the following signals, a broad doublet at δ 5.61 with *J =* 5.3 Hz corresponding to H-6. A multiplet in δ 3.59 corresponding to H-3 and the methyl groups corresponding to the proposed structure are present between δ 0.69 and 1.10. The ^13^C-NMR spectrum (CDCl_3_): δ 11.79 (C-18), 18.23 (C-19), 18.89 (C-21), 20.68 (C-11), 22 (C-16), 67 (C-26), 23.04 (C-23 and C-27), 24.28 (C-15), 28.26 (C-34 (C-2), 35.83 (C-20), 36.08 (C-22), 36.99 (C8), 37.38 (C-1), 37.49 39.15 (C-12 and C-24), 42.12 (C-4), 42.26 (C-9 and C13), 49.47 (C-14), 65.35 (C-7), 71.33 (C-3), 123.84 (C-6), and 146.23 (C-5), the data were compared with the literature [25].

#### 3β-Hydroxy-cholest-5-en-7-one and 3β-hydroxy-cholest-5,22-dien-7-one

The mixture of the 3β-hydroxy-cholest-5-en-7-one (G) and 3β-hydroxy-cholest-5,22-dien-7-one (H) steroids was identified in the UN-283 sample. The ^1^H-NMR spectrum (CDCl_3_) shows the characteristic signals of these steroids as δ 5.69 of H-6 of the two steroids: A double-doublet in δ 5.18 with *J =* 15.26, 8.85 Hz of H-23 (3β-hydroxy-cholest-5,22-dien-7-one) and a double-doublet in δ 5.03 with *J =* 15.16, 8.54 Hz of H-22 (3β-hydroxy-cholest-5,22-dien-7-one). A δ 3.68 multiplet of the H-3 of both steroids and the steroid methyl groups are present between δ 0.68 and 1.21.

The ^13^C-NMR spectrum (CDCl_3_): δ 11.96 (C-18 and C-29), 17.31 (C-19), 18.98 (C-26) (C-11), 22.69 (C-28), 23.04 (C-15), 26.31 (C-23), 28.55 (C- C-16), 29.36 (C-25), 31.18 (C-2), 33.72 (C-22), 36.08 (C-20), 36.34 (C-1), 36.27 (C-10), 38.68 (C-12), 41.79 (C-4), 43.09 (C-13), 45.41 (C-8), (C-9), 54.69 (C-17), 70.52 (C-3), 126.11 (C-6), 165.06 (C- 5), and 202.33 (C-7), for 3β-ol-choleste-5,19-dien-7-one: δ 11.96 (C-18), 12.25 (C-29) 31 (C-19), 19.03 (C-27), 19.78 (C-26), 20.49 (C-21), 21.2 ), 23.04 (C-15), 28.55 (C-16), 31.18 (C-2), 31.9 (C-25), 36.34 (C-1), 36.27 (C-10), 38.68 (C-12), 40.25 (C-20), 41.79 (C-4), 43.09 (C-13), 45.41, C-17, C-14, C-14, C-14, C-14, (C-22), 138.08 (C-22), 165.06 (C-5), and 202.33 (C-7), data were compared with the literature [26].

#### Methylanomaline

The sample named UN-244 was obtained from the Hex/CHCl_3_ and DCM/CHCl_3_ fractions, by CC, analytical thin-layer chromatography and preparative thin-layer chromatography. Methylanomaline (I) was identified by means of ^1^H-NMR and ^13^C-NMR [27] (Table-4).

**Table-4:** Chemical shifts (δ ppm) obtained from hydrogen and carbon nuclear magnetic resonance analysis of methylanomaline and anomaline isolated from the stem of *Microplumeria anomala* (Apocynaceae).

Carbon	Methylanomaline [20]					Carbon	Anomaline [20]	
	
	RMN-^1^H*	RMN-^13^C	DEPT*	RMN-^1^H**	RMN-^13^C		RMN-^1^H	RMN-^13^C
2C	3.93 m	68.71	CH	3.91 dd J=13.43. 4.88	70.05	2C	3.72 m	68.63
3C	3.16 ddd J=13.43. 13.43. 3.66	34.37	CH_2_	3.15 ddd *J=*12.51.12.5 e 3.36	35.89	3C	3.45 m	48.21
	4.03 dd J=13.42. 4.88			4.15 dd J=11.29. 6.41			4.46 dd *J=*10.68. 6.41	
5 C	2.27 *dd J=*16.78. 1.22	46.54	CH_2_	2.27 dd J=16.78. 1.27	47.31	5C	2.41 m	52.06
	2.5 d *J=*17.09			2.54 d J=16.78			2.45 m	
6 C	1.51 m	22.30	CH_2_	1.55 m	23.75	6C	1.55	38.09
	1.54 m			1.59 m			1.71 m	
7 C	-	47.40		-	47.31	7C	-	51.93
8 C	-	138.28		-	138.28	8C	-	138.55
9 C	6.74 d J=8.24	110.16	CH	6.86 d J=8.24	111.86	9C	6.88 d J=8.24	114.46
10 C	6.67 d J=8.24	112.44	CH	6.82 d J=8.24	114.68	10C	6.83 d J=7.93	112.21
11 C	-	150.		-	151.28	11C	-	156.07
12C	-	127.55		-	128.79	12C	-	151.5
13C	-	128.85		-	131.28	13C	-	128.38
14C	1.98 m	26.42	CH_2_	1.99 m	27.28	14C	1.97 m	28.68
	1.77 m			1.74 m			2.22 m	
15C	3.84 m	67.45	CH	3.76 m	68.00	15C	3.75 m	68.41
16C	1.34 m	21.17	CH_2_	1.35 m	22.22	16C	1.499 m	21.39
	1.53 m			1.60 m			1.93 m	
17C	3.93 m	23.18	CH_2_	3.91 *dd J=*13.43. 4.88	24.18	17C	2.15 m	24.31
	1.89 m			1.91 m			3.51 m	
18C	0.69 *t*	5.91	CH_3_	0.68 *t*	6.25	18C	0.701 t J=7.32	6.11
19C	1.19 dq J=14.64. 7.63	22.30	CH_2_	1.11 *dq J=*14.34. 7.63	22.76	19C	1.175 dqJ=14.66. 7.32	35.29
	1.49			1.45 m			1.599 m	
20C	-	39.29		-	40.59	20C		42.09
21C	4.00 *s*	59.97	CH	4.09 s	61.81	21C	3.94 s	66.04
2(OCH_3_	3.89 *s*	56.49	CH_3_	3.85 s	57.19	OCH_3_	3.84	57.21
CH_3_CO	2.33 *s*	22.64	CH_3_	2.38 s	23.75	CH_3_CO	2.38	22.78
CH_3_CO	-	169.48		-	172.40	CH_3_CO		172.24

#### Anomaline

The sample named UN-374 was obtained from the Hex/CHCl_3_ and DCM/CHCl_3_ fractions, by CC, analytical thin-layer chromatography and preparative thin-layer chromatography. Anomaline (J) was identified by means of ^1^H-NMR and ^13^C-NMR [27] (Table-4).

#### Loganin

Loganin was identified in the sample named UN-365, which was obtained from the 10%ACN/H_2_O fraction, by CC, analytical thin-layer chromatography and preparative thin-layer chromatography. Loganin (K) was identified by means of ^1^H-NMR and ^13^C-NMR [28] (Table-5).

**Table-5:** Data obtained from nuclear magnetic resonance analysis of loganin and loganic acid isolated from the stem of *Microplumeria anomala* (Apocynaceae). Chemical shifts δ in ppm, CD3OD, 500 MHz, and 125 MHz.

Carbon	Loganin	Reference	Carbon	Loganic acid	Reference
1C	5.28 d J=4.77	5.29 d J=4.5	1	5.28 d J=4.55	5.26 d J=4.4
3C	7.39	7.41 s	3	7.39 s	7.38 s
5C	3.11 m	3.13 m	5	3.09 m	3.10 m
6ax	1.63 m	1.64 m	6ax	1.66 m	1.66 m
6eq	2.22 m	2.25 m	6eq	2.04 m	2.03 m
7C	4.04 m	4.06 m	7	4.04 m	4.04 m
8C	1.87 m	1.89 m	8	1.88 m	1.87 m
9C	2.02 m	2.04 m	9	2.24 m	2.23 m
10C	1.09 d J=7.02	1.11 d J=6.96	10	1.09 d J=7.05	1.09 d J=6.8
1’	4.65 d J=7.93	4.67 d J=7.29	1’	4.65 d J=7.95	4.65 d J=7.8
2’	3.19 m	3.22 m	2’	3.43 – 3.18	3.41-3.17
3’	3.36 m	3.39 m	3’		
4’	-	3.31 m	4’		
5’	-	3.32 m	5’		
6’ax	3.67 m	3.69 dd J=11.94. 5.7	6’ax	3.67 dd J=9.15. 4.25	3.67 dd J=9.15. 4.25
6’eq	3.89 m	3.92 dd J=11.9. 1.74	6’eq	3.89 dd J=11.6. 1.8	3.89 dd J=11.6 1.8
OCH_3_	3.68 s	3.70 s	OCH_3_	-	-

#### Loganic acid

Loganic acid (L) was identified in the sample named UN-365, which was obtained from the 10%ACN/H_2_O fraction, by CC, analytical thin-layer chromatography and preparative thin-layer chromatography. Loganic acid was identified by means of ^1^H-NMR and ^13^C-NMR [29] (Table-5).

#### Molecules that were tentatively identified by means of liquid chromatography coupled to mass spectrometry (LC-MS) analysis

Table-6 shows the tentative identification of nine molecules isolated from samples obtained from fraction 10%ACN/H_2_O, by means of LC-MS analysis.

**Table-6:** Tentative identification of molecules isolated from fraction 10%ACN/H_2_O, obtained from *Microplumeria anomala.*

Compound	Fraction	Rt(min)	MS	MS-MS	Formula	Tentative identification	References
1	UN-361	12.3	315.04	152.68;	C_13_H_16_O_9_	Protocatechuic acid hexoside	[21]
2	UN-361	14	193	178; 134	C_10_H_10_O_4_	Ferulic acid	[22]
3	UN-361	15	403.13	371.03; 222.81; 208.78; 196.83; 190.73; 178.76; 164.64	C_17_H_24_O_11_	Secoxyloganin	[23]
4	UN-361	38.7	367.21	176.82;	C_17_H_20_O_9_	Feruloylquinic acid	[24]
5	UN-362	10.4	329.08	166.72; 151.72	C_14_H_17_O_9_	Vanillic acid hexoside	[25]
6	UN-362	13.3	315	153.69	C_13_H_15_O_9_	Protocatechuic acid-4-O-β-hexoside	[25]
7	UN-363	14.3	359.08	196.72	C_18_H_16_O_8_	Rosmarinic acid	[26]
8	UN-365	22.7	375.12	212.81; 168.74; 124.53	C_16_H_24_O_10_	Loganic acid	[27]
9	UN-365	26.6	389.08	370.88; 344.98; 208.7; 182.75	C_17_H_26_O_10_	Loganin	[27]

## Discussion

The present study aimed at the evaluation of the antibacterial activity of M. anomala against four *E. coli* strains showing different levels of virulence and resistance to antibiotics, and to determine some of the compounds occurring in the organic extract obtained from the stems of the plant. *E. coli* ATCC^®^ 25922™ that is used in the present work has been first obtained from a clinical isolate which was deposited at ATCC by the Food and Drug Administration – FDA/NIH/USA. The *E. coli* 31/1A, 35A, and 51A were previously isolated from frigates (*Fregata magnificens*) cloaca [7]. These birds visit the Alcatrazes Archipelago (24°06´S–45°41´W; 2009), São Paulo, Brazil, the place where the collection was made. According to the previous descriptions [7], the characteristics and names of each strain as given in the former manuscript are: *E. coli* 31/1A, characterized by a virulent and resistant profile, was classified in the B2 phylogenetic group, and was named O2:H7. *E. coli* 35A, characterized by a non-virulent and resistant profile, was classified in the A phylogenetic group, and was named NP. *E. coli* 51A, characterized by a virulent and non-resistant profile, was classified in the B2 phylogenetic group, and was named ONT:H7.

*E. coli* ATCC^®^ 25922™ was used in the initial screening made with more than 1300 plant extracts obtained from the Amazon Forest, which resulted in four active extracts, being EB127 one of them [13]. For a more accurate and detailed antimicrobial analysis, the three strains of *E. coli* that were obtained from frigate’s cloaca were introduced in the analysis, to verify whether the antimicrobial potential of the plant extract would be the same, or if it would be specific to each kind of micro-organism, considering their characteristics of virulence and resistance.

According to the antibacterial findings, it was possible to observe that EB127 and its fractions showed specificity in antibacterial activity, as the fraction 10%ACN/H_2_O was more active against *E. coli* ATCC and *E. coli* 35A, which is a non-virulent and resistant strain, while fraction Hex/CHCl_3_ is active against *E. coli* 51A, which is a virulent and non-resistant strain. *E. coli* 31/1A, which is a virulent and resistant strain, showed sensibility against all the fractions, particularly the two fractions mentioned before. The interpretation can be related to the chemicals present in each of the fractions.

*M. anomala* belongs to the Apocynaceae family, which is known to have compounds widely used in therapeutics, as the vinca alkaloids vinblastine and vincristine, and some cardioactive glycosides, as those occurring in *Thevetia peruviana* [30]. Bioactive iridoids and phenolic compounds can also be found in some Apocynaceae species [31]. Although few chemical studies on *M. anomala* were reported, the presence of alkaloids such as aspidocarpine, anomaline, methylanomaline, and demethoxyanomaline has been previously described in the species [27].

EB 127 was fractionated and nine fractions of different polarities were obtained. These fractions were submitted to DDA and two active fractions, named Hex/CHCL_3_ and 10%ACN/H_2_O were selected to be chemically studied due to their significant antibacterial activity against the *E. coli* strains that were used in the present evaluation. Lupeol, 3-acetyl-11-oxo-β-amyrin, and 3-acetyl-11-β-amyrin, sitosterol, and stigmasterol, 3β, 7α-dihydroxy-cholest-5-ene, 3β-hydroxy-cholest-5-en-7-one and 3β-hydroxy-cholest-5,22-dien-7-one, anomaline, and methylanomaline were isolated from fraction Hex/CHCL_3_, while protocatechuic acid hexoside [32], ferulic acid [33], secoxyloganin [34], feruloylquinic acid [35], vanillic acid hexoside [36], protocatechuic acid-4-O-β-hexoside [36], rosmarinic acid [37], loganic acid [38], and loganin [38] were identified in fraction 10%ACN/H_2_O.

The group of compounds that are reported to occur in fraction Hex/CHCL_3_, which has been active against *E. coli* 51A, the virulent and non-resistant strain, is formed by lupeol, stigmasterol, sitosterol, 3-acetyl-11-oxo-β-amyrin and 3-acetyl-11-β-amyrin, sitosterol and stigmasterol, 3β, 7α-dihydroxy-cholest-5-ene, 3β-hydroxy-cholest-5-en-7-one and 3β-hydroxy-cholest-5,22-dien-7-one, anomaline, and methylanomaline. Lupeol was isolated and tested in the DDA model against the *E. coli* strain ATCC 25922 and in the cytotoxicity models against breast and prostate cancer cell lines, showing no significant antimicrobial or cytotoxic activity. The literature provides controversial data regarding the antibacterial activity of lupeol. While some authors report the activity of this compound against Gram-positive bacteria and fungi [39], others report that the antibacterial activity of isolated triterpenes, including lupeol, expressed as MIC, is higher than 1.0 mg/mL [40]. Triterpenes were also identified in fraction Hex/CHCL_3_, as some derivatives of amyrin, sitosterol, and stigmasterol. Studies done with *Aspidosperma pyrifolium* from the *caatinga* forest, in Brazil, showed the occurrence of some alkaloids, lupeol, and amyrin, but there are no studies to prove that these isolated molecules are related to the biological activities. The Chinese plant *Ilex asprella* is commonly used as anticancer drugs, anti-flu, and anti-inflammatory drugs. Its main components are the α- and β-amyrin triterpenes [41]. Pentacyclic triterpenoids isolated from *Alstonia scholaris* also have shown antibacterial activity [42]. A sitosterol glycosylated derivative was identified in *Rauvolfia caffra* and showed some activity against *Mycobacterium tuberculosis* [43].

Alkaloids anomaline and methylanomaline were also identified in fraction Hex/CHCl_3_. Although these alkaloids have been identified in *M. anomala* before [27], no reports concerning their biological activities were found so far. Anomaline was considered the major compound present in the crude extract obtained from the stem of *M. anomala*, followed by methylanomaline, although no activity was seen against *E. coli* strains, in the DDA assay, and no antibacterial activity against was observed to any of the *E. coli* strains tested in the present work. We figured that the antimicrobial activity of fraction Hex/CHCl3 is due to a synergistic activity of the compounds that were identified in the fraction.

The group of compounds that were identified in fraction 10%ACN/H_2_O is formed by protocatechuic acid hexoside, ferulic acid, secoxyloganin, feruloylquinic acid, vanillic acid hexoside, protocatechuic acid-4-O-β-hexoside, rosmarinic acid, loganic acid, and loganin. The iridoids belong to the terpene class and can be found in more than 50 plant families. Up to 2,500 different iridoids have been cataloged so far, and they can be found in both plants and animals. Iridoids were identified to occur in several Angiosperms and have a function of eliminating herbivorous predators through the production of poisons and for having a bitter taste. Its biological properties are related to anti-inflammatory, vasoconstricting, antiviral, antitumor, and antibacterial activities [35,44]. Fraction 10%ACN/H2O is rich in phenolic compounds. Phenolic compounds usually are related to anti-inflammatory and antioxidant activities [45], and also to antibacterial activity [46]. Fraction 10%ACN/H_2_O gathers more active compounds than fraction Hex/CHCl_3_ and was found to be more active against *E. coli* 35A, which is non-virulent and resistant. Further work concerning the evaluation of the antimicrobial activity of the isolated compounds is required, but present findings have shown that the enriched fraction named 10%ACN/H_2_O is of interest in pursuing new drugs to treat resistant *E. coli*, in veterinary.

## Conclusion

EB127, an organic extract containing triterpenes, steroids, iridoids, and alkaloids, showed activity against four *E. coli* strains. Fractions obtained from EB127, named Hex/CHCl_3_, containing the triterpenes, steroids, and alkaloids, and 10%ACN/H_2_O, containing the iridoids and phenolic acids, showed a specific antibacterial activity against the virulent or the resistant strains, respectively. None of the extract or fractions have shown cytotoxicity against breast and prostate cancer cell lines. The present findings indicate that the crude extract obtained from the stem of *Microplumeria anomala* can be considered as a potential source of new veterinary antibacterial products to be used in the treatment of *E. coli* diseases. Nineteen *Microplumeria anomala* compounds were described and their occurrence is being reported in the species for the first time.

## Authors’ Contributions

LRPC collected data. MLBP, SAF, and IECD collected plant samples. VMC, RNY, LFLR and ADV analyzed data. IBS designed and managed the study, analyzed data and wrote the manuscript. All authors have read and approved the final manuscript.
